# Evaluation of standard pyrethroid based LNs (MiraNet and MagNet) in experimental huts against pyrethroid resistant *Anopheles gambiae* s.l. M’bé, Côte d’Ivoire: Potential for impact on vectorial capacity

**DOI:** 10.1371/journal.pone.0215074

**Published:** 2019-04-11

**Authors:** Welbeck A. Oumbouke, Alphonsine A. Koffi, Ludovic P. Ahoua Alou, Mark Rowland, Raphael N’Guessan

**Affiliations:** 1 Department of Disease Control, London School of Hygiene and Tropical Medicine, London, United Kingdom; 2 Institut Pierre Richet (IPR) / Institut National de Santé Publique (INSP), Bouaké, Côte d’Ivoire; Arizona State University, UNITED STATES

## Abstract

**Background:**

There is evidence from experimental hut and household studies that the entomological efficacy of long lasting pyrethroid treated nets (LLINs) is compromised in areas of pyrethroid resistance. The rapid increase in resistance intensity in African malaria vectors could further undermine the performance of these nets. The pyrethroid resistance intensity in *Anopheles gambiae* s.l. M’bé from central Côte d’Ivoire is reported to be high (> 1700 fold). Whether this translates into an increase in entomological indicators of malaria transmission needs investigation.

**Method:**

The efficacy of two long lasting insecticidal nets (LN) MiraNet and MagNet, both alpha-cypermethrin based was evaluated in experimental huts against pyrethroid resistant *Anopheles gambiae* in M’bé, central Côte d’Ivoire. All nets were deliberately holed to simulate wear-and-tear and were tested unwashed and after 20 standardized washes.

**Results:**

The entry rates of *An*. *gambiae* s.l. into huts with insecticide treated nets were 62–84% lower than entry into huts with untreated nets (p < 0.001). Exit rates of *An*. *gambiae* s.l. with unwashed MiraNet and MagNet LNs were significantly greater than with untreated nets (50–60% vs 26%) and this effect after washing 20 times nets did not decrease. Blood-feeding with both nets was significantly inhibited relative to the untreated reference net (31–55%) (p < 0.001). Washing MiraNet LN 20 times had no significant impact on protection against *An*. *gambiae* s.l. bites but it did cause a significant fall by 40% in protection with MagNet LN (p < 0.001). All insecticide treated nets induced higher mortality of *An*. *gambiae* s.l. than the untreated net (p < 0.05). The impact though significant was limited (14–30%). The personal protection against *An*. *gambiae* s.l. bites derived from all treatments was high (75–90%). The overall insecticidal effect was compromised by pyrethroid resistance and was not detectable in some treatments.

**Conclusion:**

In this area of high pyrethroid resistance intensity (over 1700 fold), both MiraNet and MagNet LNs still conferred appreciable personal protection against mosquito bites despite inducing only slightly greater mortality of pyrethroid resistant *Anopheles* mosquitoes than untreated nets. The impact is comparable to moderately intense Benin resistance area (207 fold) and Burkina Faso (over 1000 fold). This preserved level of protection plus the small but sensitive killing of mosquitoes may continue to impact vectorial capacity despite high intensity of resistance. Nevertheless, there is an obvious need for strategies and nets with novel mode of action to enhance vector control.

## Background

Insecticide treated mosquito nets and indoor residual spraying of insecticide remain the cornerstones of public health strategies for preventing malaria. These core vector control methods have contributed to the decline in malaria burden, accounting for over three-quarters of the 663 million clinical cases of malaria averted over the past 15 years in Sub-Saharan Africa [1]. Long Lasting Insecticidal Nets (LLINs) made the major contribution due to the increased ownership and use of these nets in malaria endemic areas. The estimated proportion of households in areas at risk with at least one LLIN has increased from only 2% in 2000 to 79% in 2015 [2]. Control measures based on house spraying, on the other hand, have declined in coverage. The high cost of alternative non-pyrethroid chemicals might explain the recent decline in IRS coverage from 5 to just 3% [2]. While a range of insecticides is available for use in IRS, although effectively limited by cost, there is a few classes of insecticides (pyrethroids and pyrrole) and recently an insect growth inhibitor (pyriproxyfen) approved for net treatment [3–5]. Resistance to pyrethroids is now widespread in major malaria vectors [6], thus threatening the continued effectiveness of pyrethroid-based interventions. While a resistance mitigating plan has been developed [7], options are presently limited but momentum for the development of new classes of chemistry is growing and new products may become available in the near future [8].

Although there is extensive evidence from experimental hut and household studies showing reduced entomological efficacy of insecticide treated nets against insecticide resistant vectors [9,10], there is as yet no definitive evidence of a correlation between insecticide resistance and malaria metrics. A recent study across five countries (Benin, Soudan, Kenya, Cameroon and India) has attempted to address the question of whether insecticide resistance can undermine the protective efficacy of insecticide treated nets [11] and results from one of the study sites (Benin) showed that LLINs continue to provide some protection against malaria even with highly pyrethroid resistant anopheline mosquitoes [12]. However, determination of resistance levels (high versus low) in the study was based on WHO susceptibility assay mortality and such resistance prevalence assays may give an incomplete picture of resistance [13]. It is therefore plausible that, the absence of resistance impact on bed net efficacy seen in southern Benin could be due to the fact that the resistance intensity profiles did not differ between both study arms.

The impact of resistance is difficult to demonstrate because resistance cannot be randomized [7]. Although there is no empirical data linking experimental hut data to malaria transmission indicators, mathematical models do predict an impact on transmission [14]. Hut data furthermore provides the opportunity to assess the impact of insecticide resistance on the potential for LLINs to provide individual (blood feeding inhibition) and community level protection (killing effect).

Measuring resistance intensity in *Anopheles* mosquitoes across experimental hut stations could help link the strength of resistance with the efficacy of interventions being evaluated. So far resistance intensity using adapted CDC bottle assays has been determined in local anopheline mosquitoes from only two Western African countries (Benin and Burkina Faso). In Benin areas with moderate intensity of resistance to alpha-cypermethrin (207 fold), Interceptor 1 LN, an alpha-cypermethrin based LN washed 20 times continued to inhibit blood feeding by 47% in experimental huts [15] while in Burkina Faso with higher resistance intensity to deltamethrin (over 1000 fold) it reduced feeding by 15% [16]. Mortality rates of *An*. *gambiae* in the two scenarios were low (around 20%) but greater than that with untreated nets. This suggests that LLINs would continue to provide some level of protection even when resistance is as high as that reported in Burkina Faso. Whether such limited level of control and protection is maintained across settings with similar or higher resistance intensity needs investigation.

The intensity of pyrethroid resistance in *An*. *gambiae* s.l. from the M’bé field station in central Côte d’Ivoire is among the highest ever reported (>1700 fold) [17]. The present experimental hut study was designed to investigate whether the extremely high level of resistance observed in the local anopheline mosquito population from M’bé translates into an increase in entomological indicators of malaria transmission such as mosquito survival and blood feeding rates. The performance of two LNs (MiraNet and MagNet), both alpha-cypermethrin based was therefore evaluated in M’bé experimental huts against pyrethroid resistant *An*. *gambiae* s.l. in central Côte d’Ivoire.

## Methods

### Study area

The M’bé field site is located in central Côte d’Ivoire, 40km south of Bouaké. The station is surrounded by a large rice growing valley producing year round *An*. *gambiae* s.l., mainly M form. The resistance profile of the M’bé mosquito population appears multifactorial involving target site insensitivity and increased expression of metabolic enzymes. A recent study conducted in 2016 at the M’bé field site showed over 1700 fold resistance to deltamethrin in the local *Anopheles* mosquitoes [17]. This level of resistance intensity is among the highest ever reported in African malaria vectors.

### Susceptibility tests

Bioassays were conducted using papers treated with diagnostic concentration of 0.05% alpha-cypermethrin (insecticide on the LNs). Two to three-day old adult female mosquitoes, emerged from larvae collected at M’bé field station and reared in the insectary at the Institut Pierre Richet were used for the susceptibility tests. Approximately 100 mosquitoes in batch of 25 were exposed for 1h to insecticide-treated papers and mortality was recorded 24h later.

### Experimental huts

A field trial was carried out at M’bé in experimental huts constructed to WHOPES-approved West African design [18]. The hut trial took 5 weeks (from October to November 2014), corresponding to 25 night collections per hut. The huts were made of bricks, plastered with cement, with a corrugated iron roof. The ceilings were lined with plastic sheeting and the walls were supplied with four 1-cm window slits which serve as mosquito entry points. The huts were built on a concrete pillar surrounded by water-filled moats to prevent entry of predators. Exiting mosquitoes were captured in verandah trap.

### LLINs and washing procedure

MiraNet LN is a Long Lasting net manufactured by A to Z Textile Mills, Tanzania. Alpha-cypermethrin is incorporated into 135-denier, monofilament, high-density polyethylene (HDPE) fibres, with the target dose of 4.5g/kg alpha-cypermethrin. MiraNet LN was a prototype under evaluation by WHO for recommendation at the time of the trial.

MagNet LN is a warp knitted fabric netting material containing 5.8 g/kg alpha-cypermethrin incorporated in monofilament HDPE, 150-denier manufactured by V.K.A. Polymers. MagNet LN received full WHOPES recommendation in 2011[19].

LNs were washed individually in accordance with standardized WHO Phase II washing protocols [20]. Nets were washed in 10 litres of tap water containing 2g/litre of soap (“savon de Marseille”). Each net was agitated for 3 min, left to soak for 4 min and further agitated for 3 min totalling 10 min for one washing cycle. Agitation was done by stirring the net with a wooden pole at 20 rotations per minute. Nets were rinsed using clean water and dried horizontally in the shade and subsequently stored at ambient temperature (27°C± 2°C). The regeneration interval between washes was 2 days for MiraNet LN and 1 day for MagNet LN [18].

### Experimental hut study design

The following five treatment arms were tested in experimental huts: (i) unwashed MiraNet LN, (ii) MiraNet LN washed 20 times, (iii) unwashed MagNet LN, (iv) MagNet LN washed 20 times (v) untreated 100 denier polyester net.

Treatments were randomly assigned to five experimental huts and rotated on a weekly basis according to a randomized Latin square design to account for potential bias resulting from differential hut attractiveness. Prior to the trial, the nets were artificially holed with 16cm^2^ holes (2 on each side and 1 on each end) to simulate the physical condition of damaged net in the field. At the end of a five-night rotation, the huts were thoroughly cleaned and aired for one day to prevent cross-contamination of huts from the different treatment arms. Five adult men took part in the hut trial as volunteer sleepers after informed consent. Human volunteers slept in the huts from 20.00 to 05.00 and were rotated between huts on successive nights to minimize any bias resulting from difference in individual attractiveness to host-seeking mosquitoes. Each morning, dead and live mosquitoes were collected from inside the room, under bed nets and traps using mouth-suction aspirators and torches. Mosquito collections were done on 25 nights over 5 weeks. Upon transportation to the laboratory, mosquitoes were identified to species using taxonomic keys and gonotrophic status was scored as unfed, blood fed, semi-gravid or gravid. Live female mosquitoes were held in plastic cups covered with netting and provided with 10% honey solution; mortality was recorded after 24h.

The efficacy of MiraNet and MagNet LNs was evaluated using the following entomological parameters as per WHO guidelines [21]: (i) deterrency: the percent reduction in the number of mosquitoes in treatment hut relative to control hut with untreated net; (ii) exit rate (iii) blood feeding inhibition rate: the percentage reduction in blood feeding in hut with treated net compared to hut with untreated net; (iv) percentage mortality of adult females; (v) overall insecticidal effect (as described in N’Guessan et al [9]) = 100 (Kt-Ku)/Tu where Kt is the number killed in the treated hut, Ku is the number dying in the untreated control hut, and Tu is the total number collected from the control hut [9,22,23]; (vi): personal protection: percentage reduction in mosquito biting in hut with treated net compared to hut with untreated net = [1-(number bloodfed in treatment/number bloodfed in control) x100].

### Chemical assays

The alpha-cypermethrin content of the LNs (washed and unwashed) from the five treatment arms was assessed before, after washing and after field trial based on WHO guidelines [20]. A piece of netting measuring 30cm x 30cm was cut from each of the five locations of each net. Extraction of alpha-cypermethrin was performed using the CIPAC method [24]. Alpha-cypermethrin was extracted by refluxing with xylene for 30 minutes in presence of dioctyl phthalate as internal standard and citric acid. Concentration of the insecticide was subsequently quantified by Gas Chromatography with Flame Ionization Detection (GC-FID).

### Cone bioassays on nets

Bio-efficacy of LNs (washed and unwashed) was assessed using WHO cone bioassays at two different time points: before and after field trial. Five insectary-reared *An*. *gambiae* Kisumu females aged 2–5 days were tested in four replicate cone assays on five sections of each net as per WHO guidelines at 25± 2°C and 75± 10% humidity. Knocked down mosquitoes were scored 60 min post-exposure and mortality recorded after 24 h observation period.

### Ethical permission

Ethical approval for the study was granted by the Ministry of Health in Côte d’Ivoire and the Ethical committee of the London School of Hygiene and Tropical Medicine. Written informed consent was obtained from all trial participants. Study volunteers were monitored for potential intervention-related side effects and were provided with antimalarial drug (ACTs) when tested positive for malaria. In the event that volunteers fell sick from any disease, including malaria, they were replaced until they recover and take over.

### Statistical analysis

Data were analysed using the R statistical software version 2.15.0. Proportional outcomes from the bioassays (mortality) and the hut trial (exophily, blood feeding and mortality) were analysed using generalised linear mixed models (GLMMs) with a binomial distribution and a logit link function was fitted to the data using the “lme4” package [25]. For the hut data, net type and hut were included as fixed effects and sleepers, day of mosquito collection were treated as random effects. Interactions between bednet type and washes were also included in the models. Numeric outcomes (number entering each hut, feeding and dying) were analysed using generalised linear models with a Poisson distribution. Pairwise comparisons were performed using the “multcomp” package in R [26].

## Results

### Susceptibility tests

Prior to the experimental hut trial, WHO susceptibility assays on female *An*. *gambiae* s.l. mosquitoes from M’bé to 0.05% alpha-cypermethrin-treated papers resulted in 32% mortality (n tested = 104), indicating a high frequency of resistance to pyrethroids in the study area.

### Experimental hut trial

Overall, 3614 *An*. *gambiae* s.l. females were caught in huts over the 5-week trial at M’bé (Table 1). The entry rates of *An*. *gambiae* s.l. into huts with insecticide treated nets were 62–84% lower than entry into huts with untreated nets (p < 0.001) (Table 1).

**Table 1:** Experimental hut trial results against pyrethroid resistant *An*. *gambiae* s.l.

	Untreated net	MiraNet LN 0w	MiraNet LN 20w	MagNet LN 0w	MagNet LN 20w
Total females caught	1594^a^	257^b^	582^c^	578^c^	603^c^
% Deterrency	‒	83.9	63.5	63.7	62.2
Total females exiting	419	130	336	349	343
% Exiting (95% CI)	26.3 (24.1–28.4)^a^	50.6 (44.5–56.7)^b,c^	57.7 (53.7–61.7)^c^	60.4 (56.4–64.4)^c^	56.9 (52.9–60.8)^c^
Total females blood fed	983	100	249	159	246
% Blood feeding Inhibition	‒	36.9	30.6	55.4	33.8
Personal protection %	‒	89.8	74.7	83.8	75.0
Overall insecticidal effect (%)	‒	-5.52	-2.89	3.07	-0.69

Values in the same row sharing a letter superscript do not differ significantly (p > 0.05, GLMMs)

Exit rates of *An*. *gambiae* s.l. with unwashed MiraNet and MagNet LNs were significantly greater than untreated net (50–60% vs 26%) and washing 20 times these nets did not decrease the effect (Table 1).

**Fig 1:**
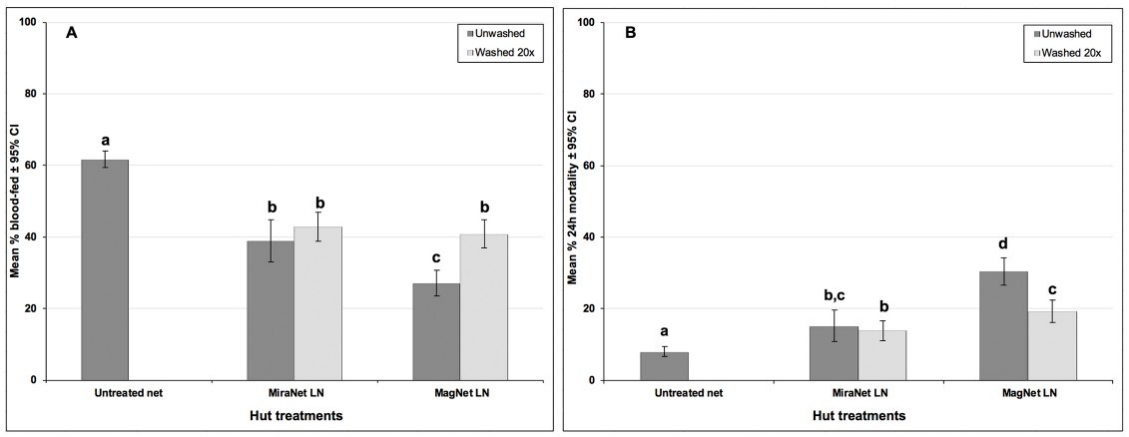
Experimental hut trial against wild free-flying pyrethroid resistant *An*. *gambiae* s.l. M’bé with MiraNet and MagNet LNs. (A) Percentage blood-feeding, (B) Percentage mortality. Bars bearing the same letter label are not significantly different at the 5% level (p < 0.05, GLMMs). Error bars represent 95% CIs.

Blood-feeding was inhibited in every hut relative to control but the levels of inhibition though significant were moderate (31–55%) (p < 0.001). Washing MiraNet LN 20 times had no significant impact on protection against *An*. *gambiae* s.l. bites, but washing MagNet LN 20 times resulted in a 40% decrease in protection, with evidence for significant interaction between net type and wash treatment (p = 0.005) (Table 1, Fig 1A).

The mortality before and after washing the LNs mirrored that of the blood-feeding. All insecticide treated nets induced higher mortality of *An*. *gambiae* s.l. than the untreated net (p < 0.05). The mortality rates across all treatment types were limited (range 14%-30%) (Fig 1B). There was evidence for significant loss of activity with MagNet LN after 20 washes but not with MiraNet LN (significant interaction between net type and wash treatment; p < 0.05).

The level of personal protection against *An*. *gambiae* s.l. bites that derived from all treatments was high (75–90%). The overall insecticidal effect on mosquitoes was compromised by pyrethroid resistance at this site and was marginal (< 4%) across all treatments (Table 1).

### Cone bioassays

Before and after field trial, knock down and mortality rates of susceptible *An*. *gambiae* s.l. were nearly 100% (> 99%) with all treated nets (data not shown).

### Chemical assays

The mean alpha-cypermethrin content in nets before and after field testing is shown in Table 2. Chemical analysis showed that initial concentrations of alpha-cypermethrin in both LNs were close to the target dose of 4.5 g/kg±25% for MiraNet LN and 5.8g/kg±25% for MagNet LN, with a within-net variation of less than 10%. After 20 washes, the alpha-cypermethrin content was 4.13 with MiraNet LN and 5.35 with MagNet LN, corresponding to an overall retention rate of about 85% for both LNs. The drop in insecticide content did not differ between MiraNet LN (14%) and MagNet LN (15%) (Table 2). While the loss in alpha-cypermethrin content after washing did not impact the efficacy of MiraNet LN (Fig 1A & 1B), the same magnitude of decline in chemical content resulted in a significant decrease in the effect size (blood feeding inhibition and mortality) with MagNet LN. After 5 weeks of use in experimental huts, there was a marginal decrease (< 10%) in alpha-cypermethrin content.

**Table 2:** Chemical analysis of alpha-cypermethrin on LNs in the experimental hut trial in M’bé.

Treatment	Concentration of alpha-cypermethrin (g/kg)		
	Before trial	After washing	After trial
MiraNet LN unwashed	4.50	–	4.62
MiraNet LN 20 washes	4.79	4.13	4.10
MagNet LN unwashed	6.43	–	5.95
MagNet LN 20 washes	6.33	5.35	4.87

## Discussion

The present study was designed to investigate in experimental huts the performance of two pyrethroid LNs (MiraNet and MagNet) against *An*. *gambiae* s.l. in an area of high resistance intensity to deltamethrin (over 1700-fold resistance) in Bouaké, Côte d’Ivoire. We observed appreciable levels of protection against mosquito bites (blood feeding inhibition) in the order of 31–55% despite high resistance intensity. These are within the protection range seen in Burkina Faso (15–25%) [27] with comparable resistance strength and in areas of lower intensity in Benin (47–57%) [15].

MiraNet and MagNet LNs in our present trial induced marginal mortality of *An*. *gambiae* s.l. (14–30%) albeit greater than the untreated nets. The trend is consistent with the hut trials from Burkina Faso and Benin and there is no evidence to suggest that increasing intensity of resistance worsen control of *An*. *gambiae* mosquitoes. However, one potential limitation of the study is that the intensity study by Glunt et al. was conducted at the same site as the current trial but at different time period: E.g. the intensity data was collected in October 2016 whereas the hut trial fell two years behind, i. e. October to November 2014. Considering that insecticide resistance is dynamic, one cannot rule out the fact that intensity might have been different at the time of the hut trial. It is plausible that with an intensity of 1700 fold in that year 2014, the corresponding effect size might have been different. Nevertheless, in the same paper by Glunt et al. PermaNet 2.0 LN, another pyrethroid-only LN, evaluated at the same site and period as the 1700-fold resistance intensity bioassays showed an impact against *An*. *gambiae* s.l. similar to that in the current trial (60% blood feeding inhibition vs 31–55%; 20% mortality vs 14–30%).

Before washing, mosquito mortality and blood feeding inhibition rates were significantly higher with MagNet LN compared to MiraNet LN. The difference in efficacy could be due to the difference in concentration of alpha-cypermethrin in both LNs (6.43 g/kg AI for MagNet LN versus 4.5 g/kg AI for MiraNet LN). While washing both nets 20 times decreased blood feeding inhibition and mortality rates, the reduction in effect size was significant only for MagNet LN, indicating that MiraNet LN was more wash resistant than MagNet LN.

Although the overall insecticidal effect of pyrethroid-treated nets is lost in the presence of resistance, a substantial protection can still be afforded to net users as evidenced in this study. Vectorial capacity as expressed by Mc Donald [28] is sensitive to the reduction in vector host contact and more so to the mortality of the malaria vector. The significant level of protection that holed nets continue to offer plus the small but sensitive killing of pyrethroid resistant anopheline population in the present study and in neighboring Benin and Burkina Faso would suggest that LLINs could still reduce malaria transmission despite resistance. This supports the WHO continuous advocacy of universal coverage with pyrethroid LNs, despite increasing level of insecticide resistance. Recent observational cohort studies conducted in Benin and Malawi demonstrated a reduction in incidence of malaria infection in LLINs users compared to bed net non-users in settings with moderate to high pyrethroid resistance [12,29]. However, this level of protection could be lost not only when resistance strength increases further [27] but also with declining bed net physical integrity [30].

To preserve the efficacy of LLINs, a range of new generation LNs have been developed. The design of these nets is generally based on the combination of unrelated insecticides (alphacypermethrin-chlorfenapyr mixture net: Interceptor G2 net) [16,31] or mixture of one insecticide with either a synergist (piperonyl butoxide-treated insecticidal net: PBO LN) [32,33] or an insect juvenile hormone mimic (permethrin-pyriproxyfen mixture net: PPF LN) [34]. Combination of insecticides with contrasting mode of action is one of the WHO recommended tactics for insecticide resistance management [7]. In a recent experimental hut trial in M’bé, Interceptor G2 LN killed very high proportion of *An*. *gambiae* s.l. (82–87%) that entered huts [35]. This effect size (high mortality) with the G2 LN demonstrates the impact of resistance on pyrethroid LNs and indicates what pyrethroids would have achieved in the absence of resistance. It also stresses the need for alternative tools or strategies to overcome insecticide resistance.

The design of new brand of bed net treated with pyrethroids only seems to be driven by the availability of commercially sustainable market further supported by the WHO policy for universal coverage with LLINs. However, with clear-cut evidence from a number of observational studies that elimination of malaria will require additional measures beyond current best practice of pyrethroid-only LNs, control effort should be devoted to the development of new and effective insecticides and strategies to counter resistance and sustain progress toward elimination.

## Conclusion

Despite high resistance intensity (over 1700 fold) found in M’bé, both MiraNet and MagNet LNs still confer appreciable protection against mosquito bites and induce slightly greater mortality of pyrethroid resistant *Anopheles* mosquitoes than untreated nets. The impact is comparable to moderately intense Benin resistance area (207 fold) and Burkina Faso (over 1000 fold). The significant level of protection that holed nets continue to offer plus the small but sensitive killing of pyrethroid resistant anopheline population would suggest that LLINs may still reduce malaria transmission despite high intensity of resistance. However, the data suggests that the community protection arising from the overall insecticidal effect of LLINs could be compromised in this area of Côte d’Ivoire with high vector resistance. There is an urgent need for development of novel strategies or LLIN with novel mode of action to enhance vector control.
